# Solid-State EDLC Device Based on Magnesium Ion-Conducting Biopolymer Composite Membrane Electrolytes: Impedance, Circuit Modeling, Dielectric Properties and Electrochemical Characteristics

**DOI:** 10.3390/membranes10120389

**Published:** 2020-12-02

**Authors:** Ahmad S. F. M. Asnawi, Shujahadeen B. Aziz, Salah R. Saeed, Yuhanees M. Yusof, Rebar T. Abdulwahid, Shakhawan Al-Zangana, Wrya O. Karim, Mohd. F. Z. Kadir

**Affiliations:** 1 Chemical Engineering Section, Universiti Kuala Lumpur Malaysian Institute of Chemical & Bioengineering Technology (UniKL MICET), Alor Gajah 78000, Malacca, Malaysia; asyafiq.asnawi@s.unikl.edu.my (A.S.F.M.A.); yuhanees@unikl.edu.my (Y.M.Y.); 2 Hameed majid Advanced Polymeric Materials Research Lab., Department of Physics, College of Science, University of Sulaimani, Qlyasan Street, Sulaimani 46001, Iraq; rebar.abdulwahid@univsul.edu.iq; 3 Department of Civil Engineering, College of Engineering, Komar University of Science and Technology, Sulaimani 46001, Iraq; 4 Charmo Research Center, Charmo University, Peshawa Street, Chamchamal, Sulaimani 46001, Iraq; salah.saeed@charmouniversity.org; 5 Department of Physics, College of Education, Old Campus, University of Sulaimani, Kurdistan Regional Government, Sulaimani 46001, Iraq; 6 Department of Physics, College of Education, University of Garmian, Kalar 46021, Iraq; shakhawan.al-zangana@garmian.edu.krd; 7 Department of Chemistry, College of Science, University of Sulaimani, Qlyasan Street, Sulaimani 46001, Iraq; wrya.karim@univsul.edu.iq; 8 Centre for Foundation Studies in Science, University of Malaya, Kuala Lumpur 50603, Malaysia; mfzkadir@um.edu.my

**Keywords:** ion conducting membrane, polymer blend, magnesium acetate, metal complex, transport study, transference number measurement (TNM) and linear sweep voltammetry (LSV) analyses, energy storage device

## Abstract

The polymer electrolyte based on Dx:Cs:Mg(CH_3_COO)_2_:Ni with three different glycerol concentrations have been prepared. The impedance study has verified that the electrolyte with 42 wt.% of glycerol (A3) has the highest ionic conductivity of 7.71 × 10^−6^ S cm^−1^ at room temperature. The ionic conductivity is found to be influenced by the transport parameters. From the dielectric analysis, it was shown that the electrolytes in this system obeyed the non-Debye behavior. The A3 electrolyte exhibited a dominancy of ions (*t_ion_* > *t_e_*) with a breakdown voltage of 2.08 V. The fabricated electrochemical double layer capacitor (EDLC) achieved the specific capacitance values of 24.46 F/g and 39.68 F/g via the cyclic voltammetry (CV) curve and the charge–discharge profile, respectively. The other significant parameters to evaluate the performance of EDLC have been determined, such as internal resistance (186.80 to 202.27 Ω) energy density (4.46 Wh/kg), power density (500.58 to 558.57 W/kg) and efficiency (92.88%).

## 1. Introduction

The studies on solid polymer electrolytes have shown an excellent performance in electrochemical devices applications such EDLCs [1,2]. These devices can provide a higher power and energy densities compared to batteries, and makes them functional in many applications such as hybrid vehicles, electronics, and voltage stabilizers [1,2]. On the other hand, with a simple and economical fabrication procedure, EDLCs have been acknowledged to offer an adequate performance which is found to be a suitable candidate to replace a conventional battery [3,4,5]. In the fabrication process of EDLCs, activated carbon is commonly chosen by researchers to build an electrode because it able to produce high conductivity value and also has large surface area [6]. Furthermore, the fermentation process of *leuconostoc mesenteroides* bacteria could grow a natural polymer called dextran (Dx), which has been recently used in the preparation of solid polymer electrolyte [7,8,9]. The various oxygen functional groups at the 1,6-α-D-glucopyranosidic linkages in Dx polymer chain helps to enhance the ionic conductivity of the system [10]. Besides, chitosan (Cs) is also one of the natural polymers that are produced from deacetylation of chitin and typically used in this field of research. The properties possessed by Cs, such as low toxicity, biodegradability and biocompatibility, make it more preferable than other polymers to be used in the preparation of solid polymer electrolyte [11,12]. Cs contains numerous functional groups such as acetamido, amino and hydroxyl groups that could form interactions with dative bonds as well as act as an electron donor [13,14]. The solid polymer electrolytes have also been widely employed in electrochemical devices because they have good film forming ability, in addition to desired mechanical and thermal characteristics [15,16]. Through polymer blending, more room will be available for the ions to hope within the long lasting solid polymer electrolytes, which result in rising the conductivity [17,18].

The ionic conductivity of an electrolyte is one of the significant factors to determine the performance of the EDLCs. Many factors have been investigated, which might affect the ionic conductivity of an electrolyte such as salt, plasticizer and metal complex. The good performance of Li^+^ ion-based salt might have few drawbacks towards the ecosystem because they are not biodegradable [19]. Consequently, some alternatives have been introduced and applied to replace the Li^+^ ion in the polymer electrolyte, such as NH_4_^+^ and Mg^2+^ ions [20,21]. Syahidah et al. [22] stated that Mg(CF_3_SO_3_)_2_ salt is suitable to be used in polymer electrolyte because it is cost effective, abundance and also easy to handle. Moreover, it was shown that the plasticized type of polymer electrolyte might experience higher ionic conductivity [23]. This hypothesis has been proven in other polymer electrolyte studies that using glycerol as the plasticizer for the system [24,25,26]. This is due to the glycerol ability to produce more ionic pathways within the electrolyte that highly impacts the performance of an electrochemical device [27,28]. In addition, the polymer electrolyte with metal complex also found to achieve higher ionic conductivity by improving the amorphous phase of the system [29]. Brza et al. [29] reported that the amorphousness of the PVA-based electrolyte was enhanced with the incorporation of Cu(II)-complex by reducing the energy band gap and described as very beneficial for the application of electrochemical devices. 

Our previous work studied the effect of Zn(II)-complex in the chitosan-NH_4_F-glycerol system and proved that the Zn metal assisted in enhancing the amorphousness of the electrolyte [30]. From the above mentioned statements, these three elements are crucial to improve the characteristics of an electrolyte especially the one that will be applied in the EDLC. In this current work, the blend of 40 wt.% Dx and 60 wt.% Cx is chosen to serve as the polymer host for this system because based on our previous work [9,31], this composition was the most amorphous blend. The different concentration of glycerol was added in the Dx-Cs based polymer electrolyte that complexed with magnesium acetate, Mg(CH_3_COO)_2_ and nickel, Ni metal. Lastly, the EDLC will be fabricated by choosing the highest conducting polymer electrolyte based on several parameters.

## 2. Experimental Details

### 2.1. Sample Preparation

All the chemical materials were bought from Sigma-Aldrich (Kuala Lumpur, Malaysia) and directly used for the sample preparation. The polymer blend host was prepared by separately dissolving 0.4 g Dx (average molecular weight 35,000–45,000) and 0.6 g Cs (average molecular weight 310,000–375,000) in 50 mL of 1% acetic acid for 90 min under an ambient temperature level. These two different solutions were mixed and stirred constantly for about 3 h at room temperature to obtain a homogeneous solution. Next, 40 wt.% of Mg(CH_3_COO)_2_ salt was added to the polymer blend solution, which then stirred at room temperature to obtain a homogeneous solution. Then, 10 mL of Ni metal was incorporated to the solutions. Subsequently, different concentration of glycerol was separately added to the electrolyte system and labeled as A1, A2 and A3 for the addition glycerol at the concentration of 14, 28, and 42 wt.%, respectively. The solutions were then casted in clean and dry labeled Petri dishes and left to dry at room temperature for films to form. Table 1 displays the composition of the electrolyte films.

### 2.2. Characterization Methods

**Figure 1:**
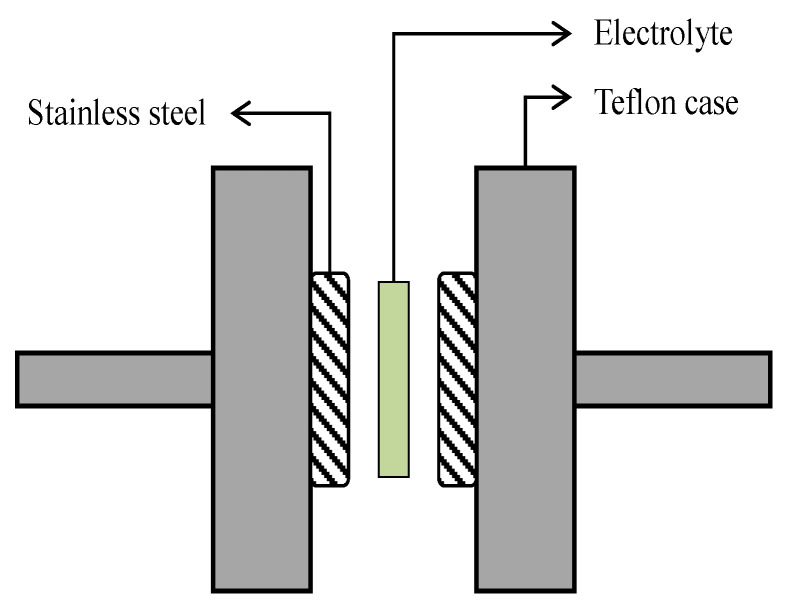
Schematic illustration of the electrolyte–electrodes arrangement for TNM and LSV studies.

The prepared electrolytes were first tested to study the impedance properties, which carried out within the frequency range of 50 Hz to 5 MHz by using a LCR meter (HIOKI 3531 Z Hi-tester, Nagano, Japan) at room temperature. From the recorded impedance data, the ionic conductivity of an electrolyte can be determined by employing the Equation (1) below.

(1) $$\sigma_{dc}=\frac{1}{R_{b}}\times \frac{t}{A}$$

where the bulk resistance, *R_b_* was determined from the intercept of the Cole–Cole plot at the *Z_r_* axis while *t* and *A* represent the thickness and surface area of the polymer composite electrolyte. Furthermore, the transference number measurement (TNM) of the ionic (*t_ion_*) and electronic (*t_elec_*) were also identified in this study by using the cell polarization plot of current against time at room temperature. The TNM study was only carried out for the highest conducting electrolyte where it was placed in a teflon holder using similar stainless steel electrodes as illustrated in Figure 1. The instrument used to conduct this measurement was the V&A Instrument DP3003 with a digital DC power supply (Shanghai, China) and the system was polarized at a working voltage of 0.20 V under an ambient temperature condition. In addition, the electrochemical stability of an electrolyte is also a significant characteristic to study. This can be obtained by using a linear sweep voltammetry (LSV) where it can record the decomposition voltage of an electrolyte at room temperature. A similar electrolyte–electrodes arrangement as in TNM study was utilized with an involvement of the highest conducting electrolyte only. A DY2300 potentiostate (Digi-Ivy, Neware, Shenzhen, China) was used to record the LSV responses towards the electrolyte at 10 mV/s. Figure 1 shows a schematic illustration of the electrolyte–electrodes arrangement for both TNM and LSV analyses.

### 2.3. EDLC Fabrication 

**Figure 2:**
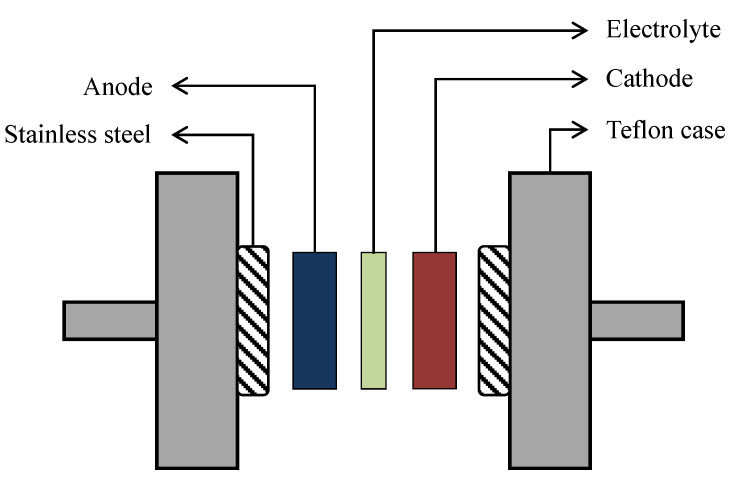
Schematic illustration of the fabricated EDLC for characterization.

The EDLC was fabricated by preparing the activated carbon (AC) electrode where 0.25 g AC was mixed with 3.25 g carbon black in the planetary ball miller. For this purpose, the powders with six metal balls were put in the chamber. The powders were mixed at rotational speed of 500 r/min for 15 min. Simultaneously, 0.50 g polyvinylidene fluoride (PVdF) was diluted in 15 mL N-methyl pyrrolidone (NMP). Then, the mixture from ball miller was poured into the PVdF-NMP solution and was stirred to obtain a thick, black solution. Consequently, an amount of acetone was used to clean an aluminum foil which then flattened on a glass surface. The prepared black solution was evenly spread on the surface of aluminum foil using doctor blade technique. It was left to dry in oven under temperature of 60 °C and then was placed in a desiccator for further drying. The arrangement of the highest conducting electrolyte and AC electrodes in the EDLC is shown in Figure 2 by using a CR2032 coin cell. 

## 3. Results and Discussion

### 3.1. Impedance Study

**Figure 3:**
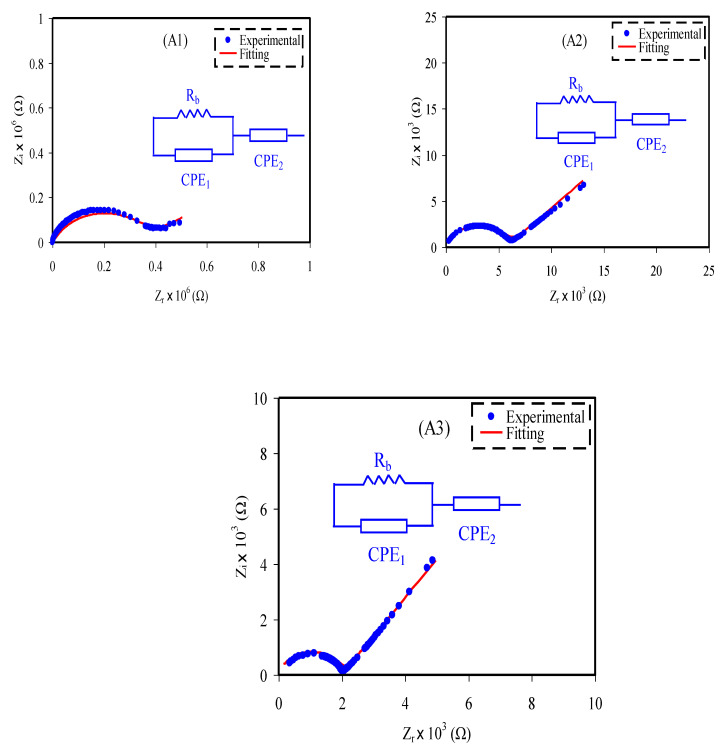
Cole–Cole plot for (**A1**), (**A2**) and (**A3**) electrolytes.

The impedance plot of the electrolyte with various glycerol concentrations at room temperature is depicted in Figure 3 where a spike and a semicircle is noted to exist at low and high frequencies, respectively [32]. The semicircle arc is due to the conduction of charges in the bulk of the electrolyte that corresponds to the parallel combination of *R_b_* and a constant phase element (CPE1), while the spike represents the accumulation of charge in polarization process that represented by CPE2, which is also a feature of a diffusion mechanism [33,34]. It is clearly detected that the semicircle in Figure 3 is getting smaller with the addition of glycerol, which attributed to prevalence of the resistive part in the electrolyte, hence causing the intercept of *R_b_* with *Z_r_* axis to decrease [35]. The impedance of CPE (*Z_CPE_*) can be expressed as [36,37]:

(2) $$Z_{CPE}=\frac{1}{C\omega^{p}}\left[\cos\left(\frac{\pi p}{2}\right)-i\sin\left(\frac{\pi p}{2}\right)\right]$$

where *C* is the capacitance of CPE, ω is the angular frequency and *p* is associated with the deviation of the plot from the axis. For the plots that consist of semicircle and spike, the *Z_r_* and *Z_i_* of the equivalent circuits can be obtained via the following equations:

(3) $$Z_{r}=\frac{R_{b}C_{1}\omega^{p_{1}}\cos\left(\frac{\pi p_{1}}{2}\right)+R_{b}}{2R_{b}C_{1}\omega^{p_{1}}\cos\left(\frac{\pi p_{1}}{2}\right)+R_{b}^{2}C_{1}^{2}\omega^{2p_{1}}+1}+\frac{\cos\left(\frac{\pi p_{2}}{2}\right)}{C_{2}\omega^{p_{2}}}$$

(4) $$Z_{i}=\frac{R_{b}^{2}C_{1}\omega^{p_{1}}\sin\left(\frac{\pi p_{1}}{2}\right)}{2R_{b}C_{1}\omega^{p_{1}}\cos\left(\frac{\pi p_{1}}{2}\right)+R_{b}^{2}C_{1}^{2}\omega^{2p_{1}}+1}+\frac{\sin\left(\frac{\pi p_{2}}{2}\right)}{C_{2}\omega^{p_{2}}}$$

where *p*_2_ and *p*_1_ are the deviation of the spike from the horizontal axis and deviation semicircle from the vertical axis, respectively. The capacitances at high and low frequency are represented as *C*_1_ and *C*_2_, respectively. The determined *R_b_* value and calculated CPE values are tabulated in Table 2.

Based on Table 2, the value of CPE is higher at low frequency compared to high frequency. This phenomenon corresponds to the following equation:

(5) $$C=\frac{\varepsilon_{o}\varepsilon_{r}A}{d}$$

where *d* is the thickness of the polymer composite electrolyte while *A* symbolizes the contact area. *ε_r_* and *ε_o_* stand for dielectric constant and vacuum’s permittivity. The value of *ε_r_* is high at lower frequency region which in turn giving a high value of *C* [38]. According to Guan et al. [39], the divalent Mg^2+^ ion has attracted towards the lone pair of oxygen atoms while the CH_3_COO^−^ anion has delocalized negative charges, which can be stabilized by the resonance stabilization and makes it as a good leaving group. This phenomenon is beneficial for the ion dissociation hence, enhance the ionic mobility and conductivity [40]. From the obtained *R_b_* values in Table 2, the ionic conductivity of the electrolytes at room temperature can be calculated using the Equation (1) as listed in Table 3. 

The addition of glycerol into the electrolyte able to improve room temperature conductivity of the current system, which optimized at the highest value of 7.71 × 10^−6^ S cm^−1^ that possessed by the A3 electrolyte. This achievement can realize the electrolyte as a promising magnesium ion conductor for the electrochemical devices applications. The ionic conductivity in this work is influenced by several variables where their correlation can be expressed as:

(6) σ=μne

where *µ*, *n* and *e* are the concentration of the mobility of ions, number density of free ions and the charge of electron, respectively [41]. The ionic conductivity increment of the electrolyte is caused by the increase in the number of *n* and *µ* and the reduction of potential barrier in the system, which correlated with the enhancement of the amorphous nature of the electrolyte. The glycerol plasticizer can dissociate more salts and disrupt hydrogen bonding between polymer chains [25,27]. Thus, this improve the overall amorphous phase of the prepared samples, which acts as a pathway for ion conduction [25,27]. Additionally, more free ions will be available for conduction. The impedance study is support this interpretation. Besides, the CH_3_COO^−^ anion will penetrate into the polymer matrix and create an attractive force between plasticizer molecules and chain segments where these forces will reduce the cohesive attraction between polymer chains that caused the segmental mobility to increase hence further enhance the ionic conductivity [42].

### 3.2. Dielectric and Electric Modulus Studies

To more understanding the polarization effects and the conductivity performance of the electrolyte system, dielectric analysis is carried out. The dielectric constant, $\varepsilon_{r}$ signifies the charge stored and the dielectric loss, $\varepsilon_{i}\;$ is the value of energy loss during the ions movement within the electrolytes [43]. The values of dielectric loss and dielectric constant can be calculated using the following expressions where the vacuum capacitance, *C_o_* is included.

(7) $$\varepsilon_{r}=\frac{Z_{i}}{\left(Z_{r}^{2}+Z_{i}^{2}\right)C_{o}\omega }$$

(8) $$\varepsilon_{i}=\frac{Z_{r}}{\left(Z_{r}^{2}+Z_{i}^{2}\right)C_{o}\omega }$$

**Figure 4:**
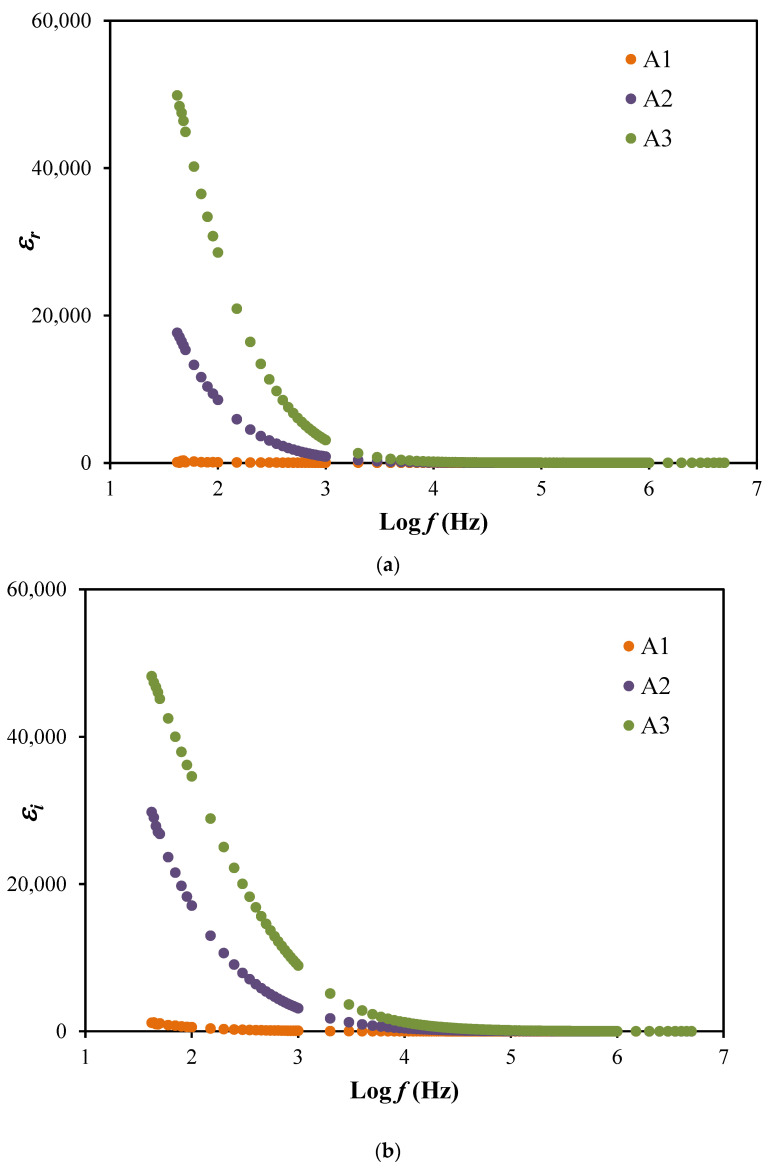
The plot of (**a**) $\varepsilon_{r}\;$ and (**b**) $\varepsilon_{i}$ for the electrolyte system.

**Figure 5:**
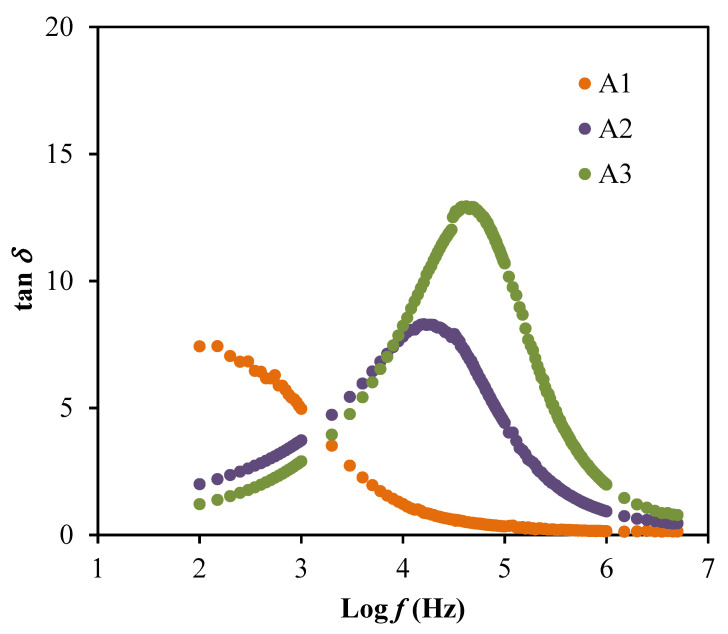
The plot of tanδ with respect to log *f*.

The plots of both dielectric properties are shown in Figure 4. The highest conducting electrolyte, A3 is observed to achieve the highest dielectric properties values at a lower frequency but then decreases as the frequency increased. This displays that the addition of glycerol may supports in enhancing the dielectric values and the number of free ions. The high values achieved from this dielectric study described the space charge effects as well as the electrode polarization that contributed by the accumulation of charge carriers [44,45]. This phenomenon explained that the electrolytes in this work obeyed the non-Debye behavior. However, the decrement of dielectric values at high frequency may due to the rapid periodic reversal of the electric field [46]. Besides, the plots of dielectric properties also did not exhibit any peaks of dielectric relaxation, which means the system is dominantly due to the polymer relaxation segments in the ionic conductivity [47]. The dielectric loss tangent (tan*δ*) is plotted as in Figure 5 to solve the relaxation processes.

The tan *δ* is a ratio of energy dissipate to energy stored in a periodical field, which is also known as dissipation factor [48]. The value of tan *δ* can be calculated this equation:

(9) $$\tan\delta =\frac{\varepsilon_{i}}{\varepsilon_{r}}$$

**Figure 6:**
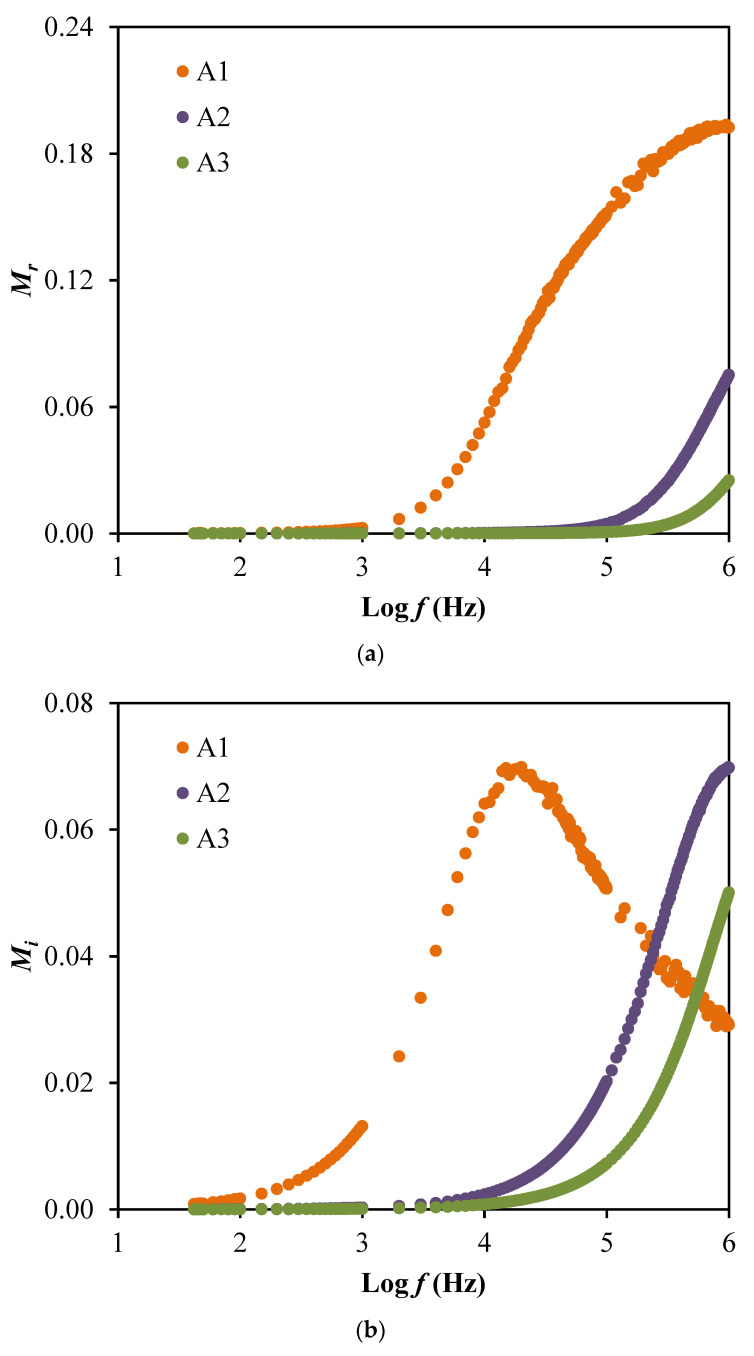
The plot of (**a**) *M_r_* and (**b**) *M_i_* for the electrolyte system.

Based on Figure 5, the peaks observed are explained as the translational ion dynamics that related to the mobile ions conductivity relaxation which further explained the decrease in segmental motion within the polymer electrolyte that beneficial to support the transportation of ions [49]. The broad tanδ peaks in Figure 5 also signifies that the relaxation process is following non-Debye behavior [50]. Furthermore, the polarization suppression effect of the system can be analyzed through the electrical modulus as plotted in Figure 6. The real part (*M_r_*) and imaginary part (*M_i_*) of electrical modulus can be determined using these relations:

(10) $$M_{r}=\frac{\varepsilon_{r}}{\varepsilon_{r}^{2}+\varepsilon_{i}^{2}}$$

(11) $$M_{i}=\frac{\varepsilon_{i}}{\varepsilon_{r}^{2}+\varepsilon_{i}^{2}}$$

At low frequencies, *M_r_* and *M_i_* approach to zero because the electrode polarization is dominant and no dispersion is observed [31]. Long tail at low frequencies indicates the capacitive behavior. The highest conducting electrolyte will produce the lowest *M_r_* and *M_i_* values at a high frequency. The presence of peaks in both modulus plots explain that the electrolytes are good ionic conductors at higher frequencies [51]. 

### 3.3. Transport Study

Based on Equation (6), two significant parameters, *n* and μ, are important to influence as well as support the ionic conductivity of the electrolytes. According to Arof et al. [52], these ionic transport parameters can be evaluated using the electrical impedance spectroscopy (EIS) approach by fitting the impedance curve in Figure 3. This method expressed diffusion coefficient, *D* using the Equation (12) for the Cole–Cole plots with semicircles and spikes.

(12) $$D=\frac{{\left(k_{2}\varepsilon_{o}\varepsilon_{r}A\right)}^{2}}{\tau_{2}}$$

The Equation (12) required the $\tau_{2}$, which represents the inverse of ω  at the minimum value of *Z_i_*. Fadzallah et al. [53] mentioned that $\varepsilon_{r}$ was taken from a constant value of log $\varepsilon_{r}$ in the transport study, while Yusof et al. [54] mentioned in their report that $\varepsilon_{r}$ was taken at log *f* > 5 where the values were almost constant. The μ values can be determined by substituting Equation (12) into the following equation:

(13) $$\mu =\frac{eD}{k_{B}T}$$

where *k_B_* and *T* are the Boltzmann constant and an absolute temperature, respectively. Table 4 provides the values of *p*_2_, *k*_2_, *ε_r_* and *τ*_2_ for the electrolytes in this work.

By using the Equations (6), (12) and (13), the transport parameter can be obtained as listed in Table 5 for the electrolytes in this work. The trend of the transport parameters are harmonized with the ionic conductivity pattern where the addition of glycerol helped to increase the values and the highest conducting electrolyte possesses the highest transport parameters values. Pritam et al. [55] also reported the similar trend where the increase in transport parameter values were possibly related to the low degree of crystallinity of an electrolyte. Thus, this would improve the segmental motion within the polymer chain, which therefore the electrolyte would achieve a high ionic conductivity [55].

### 3.4. Transference Number Measurement (TNM)

**Figure 7:**
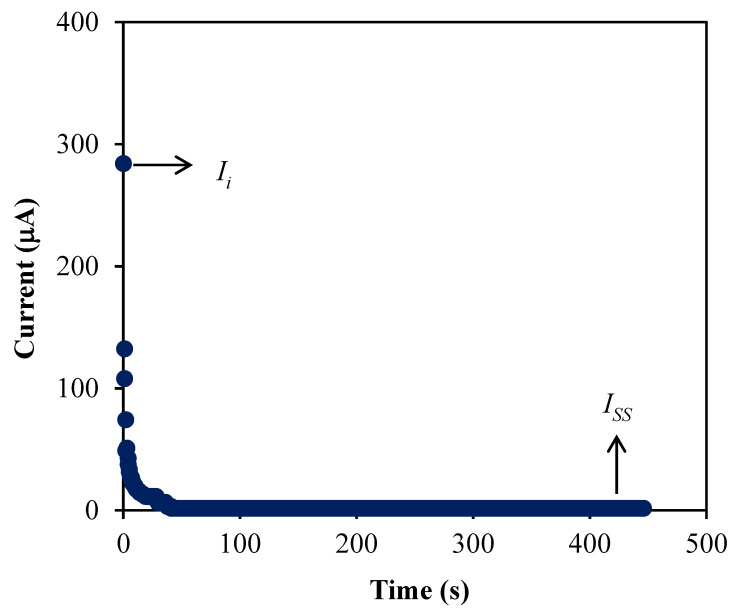
The plot of current versus time for the A3 electrolyte.

To determine which charge carrier species is dominant within the electrolytes, a measurement called TNM was conducted in this work. Figure 7 shows the plot of current versus time for the A3 electrolyte. The electrolyte is mostly due to ions if the ionic transference number, *t_ion_* value is larger than the transference number of electron, *t_e_* where these both values can be determined using the following expressions that initial current, *I_i_* and steady state current, *I_ss_* are involved [56].

(14) $$t_{ion}=\frac{I_{i}-I_{ss}}{I_{i}}$$

(15) $$t_{e}=\frac{I_{ss}}{I_{i}}$$

Based on the polarization plot in Figure 7, the *I_i_* is noticed to drastically decrease as the time increased. This is because the ionic species in the electrolyte is dropped at this stage and leading towards *I_ss_* when the species is completely reduced [57]. Besides, the decrement of ionic species is caused by the blockage of ions flows due to stainless steel electrodes that only allow the electron to move [58]. The diffusion process is beneficial at *I_ss_* to balance the mobile ions movements [59]. In this study, the A3 electrolyte achieved a high *t_ion_* value which is 0.9936 while the *t_e_* is 0.0064. This result has signified that the electrolyte consists of more ions compared to electrons and also agreed with other studies [30,60].

### 3.5. Linear Sweep Voltammetry (LSV) Study

**Figure 8:**
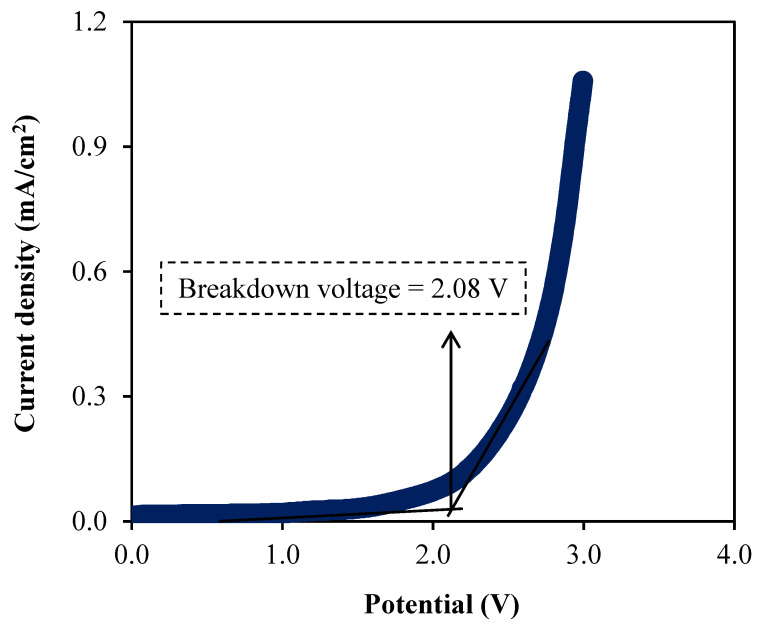
The plot of LSV curve for the A3 electrolyte.

The LSV study is carried out to reveal the breakdown voltage of an electrolyte at room temperature [61]. Figure 8 depicts the LSV plot up to 4.0 V for the A3 electrolyte.

The plot of current density versus voltage as shown in Figure 8 reveals the raise of voltage without any current flows involved until 2.08 V, which explain that the reaction of electrochemical in the electrolyte did not occur at this stage [62]. This phenomenon indicates that the breakdown voltage of the electrolyte is at 2.08 V, which is comparable as reported in the literature [63]. Hence, this result shows that the ability of this electrolyte to deliver a promising performance in the EDLC that is normally run at the operating voltage of 1.0 V [64].

### 3.6. Characterization of the EDLC

**Figure 9:**
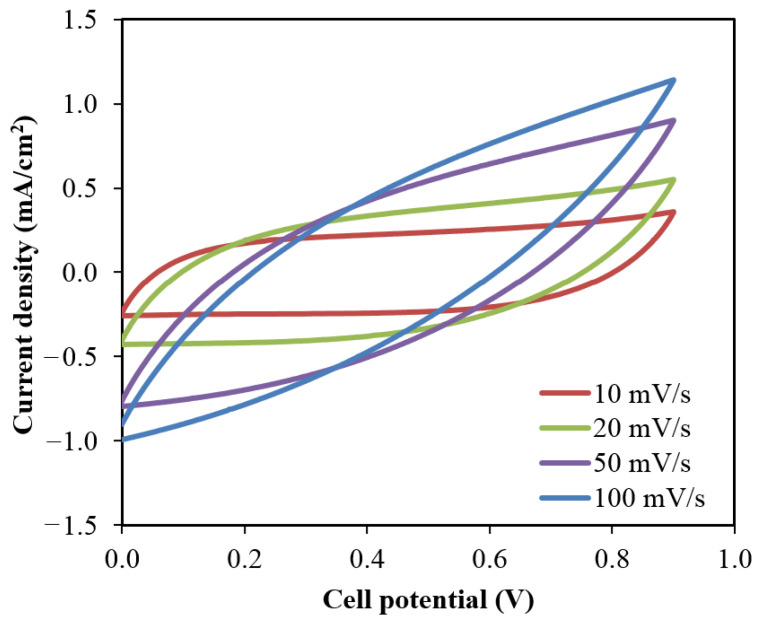
The plot of CV curves at different scan rates.

The cyclic voltammetry (CV) measurement is one of the crucial analyses to identify the charge storage and charge transfer behaviors in the fabricated EDLCs. This measurement was conducted using a pentiostat (Digi-IVY DY2300) (Neware, Shenzhen, China) at different scan rates, *v* as shown in Figure 9. The specific capacitance from CV analysis can be calculated via the following equation where the area of the CV curve, *I(V) dV* and the mass of active materials, *m* are involved [65]. The voltage range for CV measurement was between 0.0 V (*V*_1_) to 0.9 V (*V*_2_).

(16) $$C_{s}=\int_{V_{1}}^{V_{2}}\frac{I\left(V\right)dV}{2mv\left(V_{2}-V_{1}\right)}$$

The CV curves in Figure 9 exhibit that as the scan rate increases, the shape of the curve changes from rectangular shape to a leaf-like shape. These changes are caused by the existence of the porosity of the carbon electrodes and the internal resistance presents during the measurement [66]. The curves also depicted without any noticeable peaks that further explained the system did not undergo any reduction/oxidation reaction. The anions within the system will flow towards the positive electrode during the charging process where the positive electrode will repel the cations so that the cations will attracts to the negative electrode. The electric field during this process embraces the electrode and electrolyte to hold the electrons and ions, respectively [67]. These processes are the explanation for the formation of double-layer charge to store the potential energy on the carbon electrodes surfaces [68]. The calculated specific capacitance, Cs from CV curves analysis is listed in Table 6 at various scan rates.

**Figure 10:**
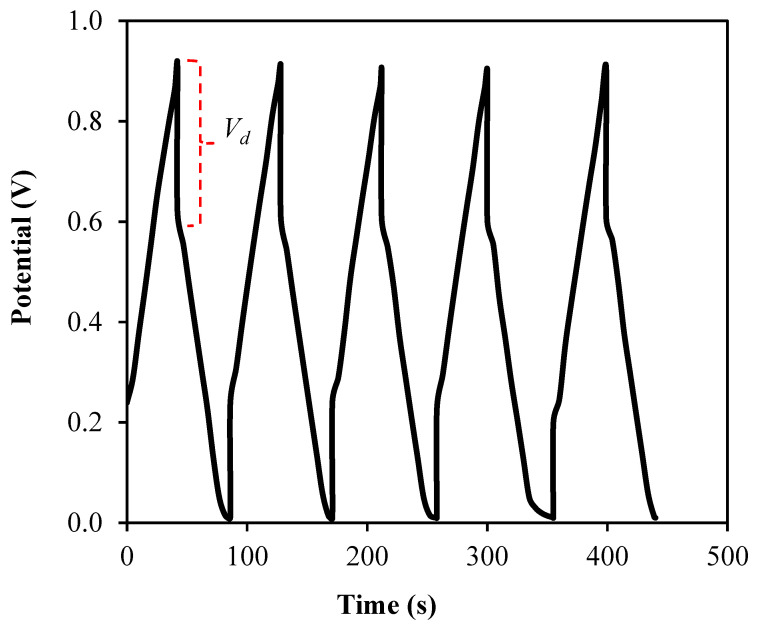
Charge–discharge curves at the selected cycles.

**Figure 11:**
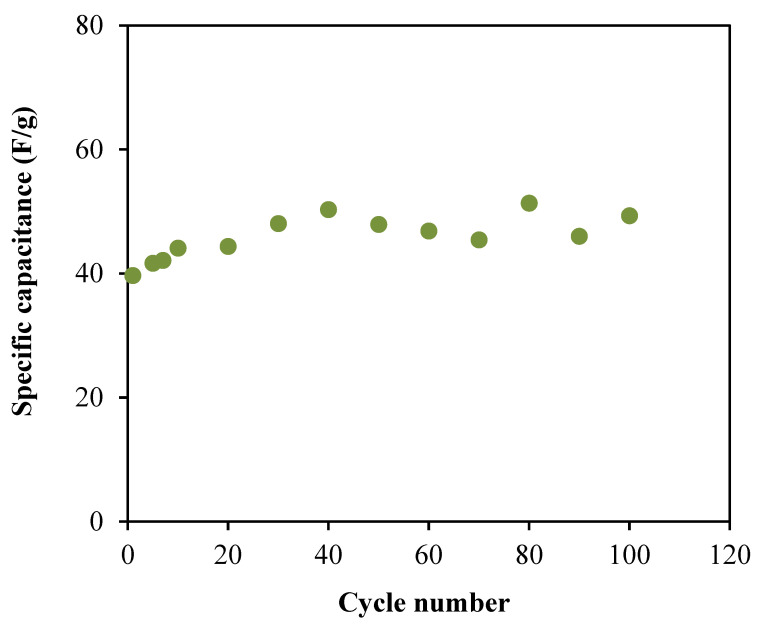
Specific capacitance, *C_s_* of the fabricated EDLC.

The low *C_s_* value at higher scan rate is because the energy loss is high at these scan rates due to the reduction of the amount of stored charges, which exhibits lower *C_s_* value [69]. Further characterization of the fabricated EDLC involved the charge–discharge profile, as shown in Figure 10, using the Galvanostatic technique by employing a Neware battery cycler. This profile is valuable to determine the cyclic durability of the system as well as their capacitive behavior. The capacitive behaviour in the EDLC is comfirmed when the discharge slope is near to a straight line or linear curve [70]. As a comparison to the *C_s_* values from CV studies, the *C_s_* values also can be calculated from the profile in Figure 10 by using the equation below and the obtained values are plotted in Figure 11.

(17) $$C_{s}=\frac{i}{ms}$$

The calculated Cs value for the first cycle is 39.68 F/g, which is slightly higher than the values obtained from CV analysis. This difference is reliable because both analyses display the capacitive properties of the EDLC [71]. At the 40th cycle, Cs value is increased to 50.34 F/g and then maintained at an average of 48.19 F/g until the system completed the 100 cycles. The achievement based on the satisfactory specific capacitance values obtained by the EDLC, it can be concluded that the electrolyte in this work is suitable enough to be applied in the electrochemical devices applications. Moreover, a tiny voltage drop, *V_d_* is noticed in the charge–discharge profile as shown in Figure 10. The existence of *V_d_* can be attributed to the internal resistance within the system during the charge–discharge processes, which also called as equivalent series resistance (ESR). The determination of this parameter will involve the applied current, *i* of 1.5 mA and can be expressed as:

(18) $$ESR=\frac{V_{drop}}{i}$$

**Figure 12:**
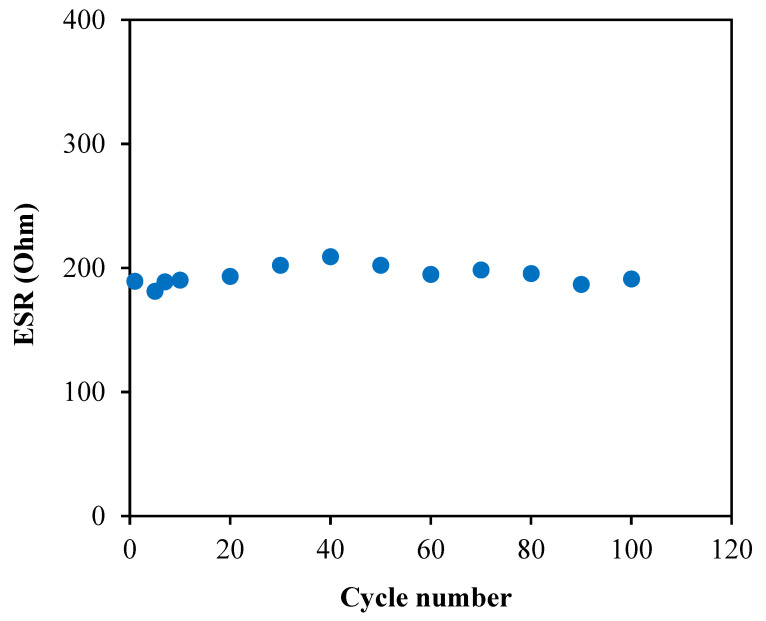
Equivalent series resistance, ESR of the fabricated EDLC.

The obtained ESR values for 100 cycles are illustrated in Figure 12, where 189.33 Ω is observed at the first cycle. Throughout the whole processes, the system is noticed to maintain its ESR at the range of 186.80 to 202.27 Ω. This result shows that the EDLC has a small and constant internal resistance for 100 cycles which simplifies the electrostatic process between the ions and charged electrode [72].

**Figure 13:**
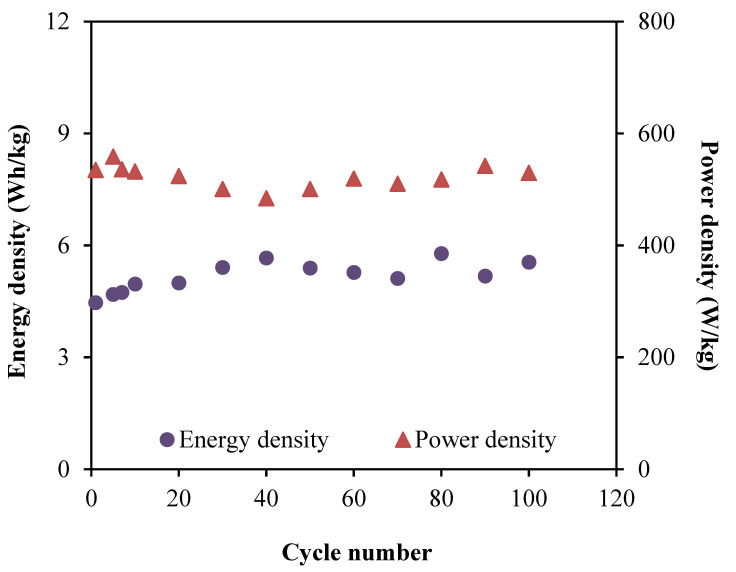
Energy density and power density of the fabricated EDLC.

Figure 13 depicts the calculated energy and power densities of the EDLC for 100 cycles. The values are determined from the Equations (19) and (20), where the operating voltage value, *V,* is included, which is at 0.9 V.

(19) $$E=\frac{C_{s}V^{2}}{2}$$

(20) $$P=\frac{V^{2}}{4m\left(ESR\right)}$$

**Figure 14:**
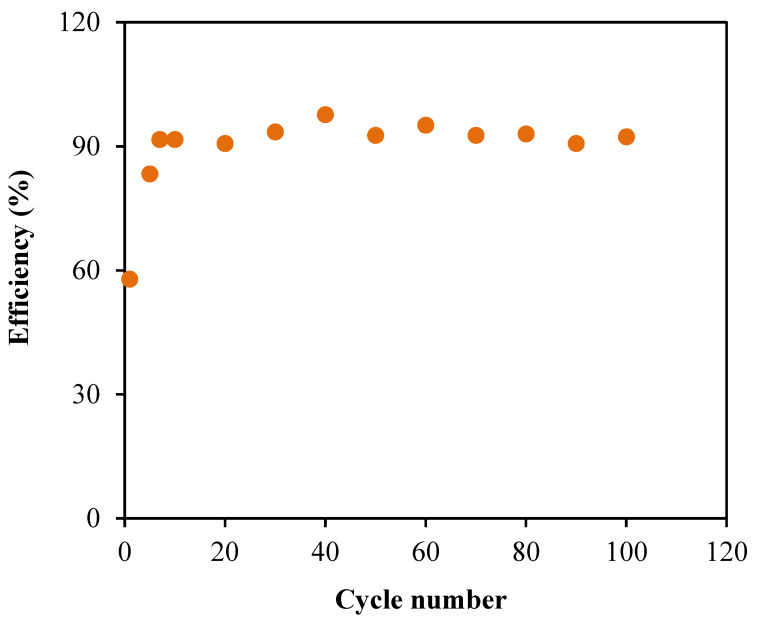
The efficiency η of the fabricated EDLC.

Based on Figure 13, the EDLC in this work achieved the energy density of 4.46 Wh/kg at the first cycle. Then, the energy density is steadily increased to 5.66 Wh/kg (40th cycle), which remained around 5.42 Wh/kg until the cycle is completed. The pattern of energy density agrees with the specific capacitance trend in Figure 11, which described that the required amount of energy for the charge carriers in each cycle of the charge–discharge process is almost equivalent in order to move towards the electrodes surfaces [73]. On the other hand, the plot of power density in Figure 13 shows a comparable trend as the ESR plot. The range of power density for the fabricated EDLC in this work is between 500.58 to 558.57 W/kg. The almost-consistent values throughout the 100 cycles clarifies that ELDC possesses the capacitive characteristics [74]. This also means that the electrolyte consists of enormous amount of free ions, which contributed to the formation of a double layer [75]. Lastly, the performance of EDLCs can be analyzed using the Columbic efficiency, η where the stability of each cycle can be revealed as plotted in Figure 14. This parameter involves the time of discharging, *t_d_* as well as charging, *t_c_*, which can be expressed using the following equation:

(21) $$\eta =\frac{t_{d}}{t_{c}}\times 100$$

The fabricated EDLC has a low efficiency of 57.89% at the first cycle. However, the performance is found to enhance for the next cycle until the EDLC completed the 100 cycles where the efficiency values are consistent at an average of 92.88%. This result suggests that the system presents an excellent performance in electrochemical devices with good contact of electrolyte–electrode because the efficiency is above 90.0% [76].

## 4. Conclusions

The polymer electrolytes based on Dx:Cs:Mg(CH_3_COO)_2_:Ni with three different glycerol concentration have been well-prepared. The impedance study has verified that the electrolyte with 42 wt.% of glycerol (A3) has the highest ionic conductivity of 7.71 × 10^−6^ S cm^−1^ at room temperature. The ionic conductivity is found to be influenced by the transport parameters. The understanding from dielectric analysis revealed that the electrolytes in this system obeyed the non-Debye behavior. The A3 electrolyte exhibited a dominancy of ions (*t_ion_* > *t_e_*) with a breakdown voltage of 2.08 V. The fabricated EDLC achieved the specific capacitance values of 24.46 F/g and 39.68 F/g via CV curve and the charge–discharge profile, respectively. The other significant parameters to evaluate the performance of EDLC have been determined, such as internal resistance (186.80 to 202.27 Ω), energy density (4.46 Wh/kg), power density (500.58 to 558.57 W/kg) and efficiency (92.88%). These results imply that the system presents an excellent performance in some electrochemical devices with a good contact of electrolyte–electrode because the efficiency is above 90.0%.

## Figures and Tables

**Table 1:** Designation of the polymer electrolytes with different amount of glycerol.

Glycerol (wt.%)	Designation
12	A1
28	A2
42	A3

**Table 2:** The parameter of circuit element for the electrolyte system with different glycerol concentration at room temperature.

Electrolyte	$R_{b}\;(\Omega )$	CPE1 (F)	CPE2 (F)
**A1**	3.00 × 10^5^	5.00 × 10^−10^	5.88 × 10^−7^
**A2**	5.80 × 10^3^	2.50 × 10^−9^	5.56 × 10^−6^
**A3**	2.00 × 10^3^	3.33 × 10^−9^	6.06 × 10^−6^

**Table 3:** Ionic conductivity for the electrolyte system with different glycerol concentration at room temperature.

Electrolyte	Ionic Conductivity, σ (S cm^−1^)
**A1**	5.14 × 10^−8^
**A2**	2.66 × 10^−6^
**A3**	7.71 × 10^−6^

**Table 4:** The calculated values of *p*_2_, *k*_2_, *ε_r_* and *τ*_2_ for the electrolyte system.

Electrolyte	*p*_2_	*k*_2_ (F^−1^)	$\varepsilon_{r}\;$	$\tau_{2}(s)$
**A1**	0.38	1.70 × 10^6^	4.76	2.65 × 10^−4^
**A2**	0.49	1.80 × 10^5^	10.64	5.89 × 10^−6^
**A3**	0.60	1.65 × 10^5^	12.72	4.86 × 10^−6^

**Table 5:** The calculated transport parameters for the electrolyte system.

Electrolyte	*n* (cm^−3^)	*μ* (cm^2^V^−1^s^−1^)	*D* (cm^2^s^−1^)
**A1**	5.20 × 10^19^	6.17 × 10^−9^	1.58 × 10^−10^
**A2**	2.32 × 10^21^	7.14 × 10^−9^	1.83 × 10^−10^
**A3**	5.50 × 10^21^	8.75 × 10^−9^	2.25 × 10^−10^

**Table 6:** Specific capacitance, *C_s_* of the EDLC at different scan rates.

Scan Rate (mV/s)	Specific Capacitance (F/g)
**100**	4.18
**50**	8.46
**20**	17.15
**10**	24.46
